# Antidepressant fluoxetine alleviates colitis by reshaping intestinal microenvironment

**DOI:** 10.1186/s12964-024-01538-5

**Published:** 2024-03-12

**Authors:** Shuo Teng, Yi Yang, Wanru Zhang, Xiangji Li, Wenkun Li, Zilu Cui, Li Min, Jing Wu

**Affiliations:** 1 Department of Gastroenterology, Beijing Friendship Hospital, Capital Medical University, National Clinical Research Center for Digestive Disease, Beijing Digestive Disease Center, Beijing Key Laboratory for Precancerous Lesion of Digestive Disease, Beijing, 100050 People’s Republic of China; 2 Peking University Ninth School of Clinical Medicine, Beijing, 100038 China

**Keywords:** IBD, Single cell sequencing, Intestinal microenvironment

## Abstract

**Background:**

The impact of antidepressants on Inflammatory bowel diseases (IBD) has been extensively studied. However, the biological effects and molecular mechanisms of antidepressants in alleviating colitis remain unclear.

**Methods:**

We systematically assessed how antidepressants (fluoxetine, fluvoxamine and venlafaxine) affected IBD and chose fluoxetine, the most effective one, for mechanism studies. We treated the C56BL/6 mice of the IBD model with fluoxetine and their controls. We initially assessed the severity of intestinal inflammation in mice by body weight loss, disease Activity Index scores and the length of the colon. The H&E staining and immunohistochemical staining of MUC2 of colon sections were performed to observe the pathological changes. RT-qPCR and western blot were conducted to assess the expression level of the barrier and inflammation-associated genes. Then, single-cell RNA sequencing was performed on mouse intestinal mucosa. Seurat was used to visualize the data. Uniform Manifold Approximation and Projection (UMAP) was used to perform the dimensionality reduction. Cell Chat package was used to perform cell–cell communication analysis. Monocle was used to conduct developmental pseudotime analysis. Last, RT-qPCR, western blot and immunofluorescence staining were conducted to test the phenomenon discovered by single-cell RNA sequencing in vitro.

**Results:**

We found that fluoxetine treatment significantly alleviated colon inflammation. Notably, single-cell RNA sequencing analysis revealed that fluoxetine affected the distribution of different cell clusters, cell–cell communication and KEGG pathway enrichment. Under the treatment of fluoxetine, enterocytes, Goblet cells and stem cells became the dominating cells. The pseudotime analysis showed that there was a trend for M1 macrophages to differentiate into M2 macrophages. Lastly, we tested this phenomenon in vitro, which exhibited anti-inflammatory effects on enterocytes.

**Conclusions:**

Fluoxetine exhibited anti-inflammatory effects on intestinal mucosa via remodeling of the intestinal cells and macrophages, which reveals that fluoxetine is a promising therapeutic drug for the treatment of IBD and psychiatric comorbidities.

**Supplementary Information:**

The online version contains supplementary material available at 10.1186/s12964-024-01538-5.

## Background

As a chronic long-term digestive disease, inflammatory bowel disease (IBD) causes a significant medical burden on patients worldwide [1]. Even though there are a lot of therapeutics invented for IBD, there is still a ceiling to the curative effects to bring about complete mucosal healing. Thus, IBD is usually refractory, and a large portion of patients with IBD experience clinical relapse after being clinically cured. There are significant signs of pathology in the gut microenvironment and distant organs in IBD patients, such as the disturbed brain-gut axis, which partially explains the “therapeutic ceiling” of IBD. Therefore, an in-depth investigation of both the mucosal pathology and change of the brain-gut axis during IBD initiation and progression is crucial for IBD drug development.

Noticeably, there is a high frequency of psychiatric comorbidities closely associated with IBD. The recurrent and lifelong symptoms may render IBD patients vulnerable to the development of mental illness [2, 3]. There are up to one-third of IBD patients experienced anxiety and one-fourth experienced depression [4, 5]. Additionally, patients with a history of depression were more likely to be diagnosed with IBD [6–8]. Some clinicians further found that the use of antidepressants in the treatment of depression may diminish the increased risk of IBD caused by depression [9]. However, it is still unclear which antidepressants are beneficial and how they function. It would be engaging and necessary to investigate the potential mechanisms underlying this clinical phenomenon.

Antidepressants include selective serotonin reuptake inhibitors (SSRIs), tricyclics, and, monoamine oxidases [10], and SSRIs are the most commonly prescribed antidepressant medications. The anti-inflammatory effects of SSRIs can be divided into two categories according to the mediators of their mechanisms: central nervous system mediated mechanisms, and peripheral mechanisms promoted by tissue-resident cells or circulating immune system cells [11–14]. However, the anti-inflammatory effect of SSRIs on IBD and related mechanisms is not clear. Here, we systematically assessed how antidepressants affected IBD and chose fluoxetine, the most effective one, for mechanism studies. Through the experiments in vivo and in vitro and single-cell analysis, we found that fluoxetine could alleviate colitis by reshaping the microenvironment.

## Methods

### Animals

C57BL/6 male mice were purchased from Beijing Weitong Lihua company. All mice were raised in a 12-h day and night cycle environment, with free water and diet. The design and implementation of animal experiments have been approved by the Experimental Animal Center of Beijing Friendship Hospital [Grant No.22–2031].

DSS-induced colitis and administration of antidepressants.

Antidepressants including fluoxetine (Mecklin, F830634), venlafaxine (Mecklin, V831522) and fluvoxamine (Mecklin, F875839) were dissolved in PBS (10 mg/kg/d). Mice in the DSS + antidepressants group were given relevant antidepressant suspension intraperitoneally for 3 weeks. The vehicle group was injected intraperitoneally with PBS for 3 weeks, with a daily dose of 100ul. 7 days before harvesting, these groups above were given 3% (wt/vol) DSS (Sigma, Lot#BCCC5047) in water and drinking freely to induce colitis. Mice in the control group were fed with a normal-free diet and water for 3 weeks. Colon tissues were harvested for further pathological analysis after 3 weeks. The state of colon was scored blindly by a Disease Activity Index (DAI) scores based on the previous study [15].

### Cell culture

Human Colon cancer (CC) cell lines (Caco-2 and HT29), and human monocyte THP-1 were obtained from Cell Bank of Chinese Academy of Sciences. Caco-2 and HT29 were cultured in DMEM medium with 10% Gibco fetal bovine serum (Thermo Fisher, cat. 10,100,147, Waltham, MA, USA). THP-1 was cultured in RPMI 1640 medium with 10% Gibco fetal bovine serum. All of them were cultured at 37℃ with 5%CO2. Cells in our experiments have passed STR analysis.

### Histological analysis

Colon tissues were sectioned at 5 mm and the sections were stained with hematoxylin and eosin (H&E) (Solarbio, Beijing, China). Then the H&E sections were blindly scored by a certificated pathologist through image acquisition on a microscope. To count colonic goblet cells, intestinal Sects. (5 mm) were subjected to immunohistochemical staining to assess the expression levels of MUC2.

### Immunofluorescence staining

Immunofluorescence (IF) staining for ZO-1 expression in the colon tissues was as follows: Colon tissues were sectioned at 5 mm. After blocking under normal goat serum for 1 h, rabbit anti-ZO-1(diluted at 1:200, proteintech) was applied in these sections at 4℃ overnight. After washing 3 times in PBS, the sections were incubated in Alexa fluor® 594 conjugated goat anti‐rabbit IgG for 1 h at room temperature. Lastly, the sections were stained with DAPI and detected under the fluorescence microscope.

IF staining for ZO-1 expression in the Caco2 was as follows:

The cells were washed three times with PBS, and then treated with 0.3% Triton X-100 in PBS. After washing three times with PBS, rabbit anti-ZO-1(diluted at 1:2000, proteintech) was incubated at 4℃ overnight. Secondary antibodies were diluted to 1:200, and incubated for 1 h at room temperature. Nuclei were stained with DAPI.

### Western blot assays

Protein obtained from animal tissues or cells were quantified by BCA assay Kit Pierce BCA Protein Assay Kit (Thermo Fisher, cat. 23,225, Waltham, MA, USA), protein samples were loaded on 10% SDS-PAGE gel, transferred to a PVDF membrane (Millipore, cat. IPVH00010, MA, USA) after separation and then blocked in 5% non-fat milk. The membranes were incubated in primary antibodies overnight at 4℃, followed by specific secondary antibodies for 2 h at room temperature. The primary antibodies including anti-ZO-1 (1:1000, proteintech), anti-Occludin (1:1000, proteintech), and anti-β-tubulin (1:1000, proteintech) were used.

Reverse transcription-quantitative polymerase chain reaction (RT-qPCR).

Total RNA from colon tissue was isolated by TRIzol Reagent (Thermo Fisher, cat. 15,596,026, Waltham, MA, USA). RNA was further processed by reverse transcriptase kit (Takara, cat. RR036A-1, Tokyo, Japan). qPCR was performed by PowerUp^TM^SYBR Master Mix reagents (Life Technologies, cat. A25742, CA, USA). The expression level of mRNA was analyzed using 2^−△△CT^ standardized to GAPDH. The sequences of primers are listed in Supplementary Table 1.

**Table 1:** Sequences of primers for qPCR

mTNF-α-F	CATCTTCTCAAAATTCGAGTGACAA
mTNF-α-R	TGGGAGTAGACAAGGTACAACCC
mlL-1β-F	CCGTGGACCTTCCAGGATGA
mlL-1β-R	GGGAACGTCACACACCAGCA
mClaudin-F	TCCTATAAATCCACGCCGGTTC
mClaudin-R	CTCAAAGTTACCACCGCTGCTG
mMUC2-F	AGGGCTCGGAACTCCAGAAA
mMUC2-R	CCAGGGAATCGGTAGACATCG
mIL-6-F	ATCCAGTTGCCTTCTTGGGACTGA
mIL-6-R	TTGGATGGTCTTGGTCCTTAGCCA
miNOS-F	CACCAAGCTGAACTTGAGCG
miNOS-R	CGTGGCTTTGGGCTCCTC
mMCP1-F	CTCACCTGCTGCTACTCATTC
mMCP1-R	TTACGGCTCAACTTCACATTCA
mARG1-F	CCAGAAGAATGGAAGAGTCAGTGT
mARG1-R	GCAGATATGCAGGGAGTCACC
mCD206-F	CTGCAGATGGGTGGGTTATT
mCD206-R	GGCATTGATGCTGCTGTTATG
mIL-10-F	ACTGGCATGAGGATCAGCAG
mIL-10-R	CTCCTTGATTTCTGGGCCAT
hTNF-α-F	GAGGCCAAGCCCTGGTATG
hTNF-α-R	CGGGCCGATTGATCTCAGC
hlL-1β-F	TTCGACACATGGGATAACGAGG
hlL-1β-R	TTTTTGCTGTGAGTCCCGGAG
hlL-6-F	ACTCACCTCTTCAGAACGAATTG
hlL-6-R	CCATCTTTGGAAGGTTCAGGTTG
hCD163-F	GCGGGAGAGTGGAAGTGAAAG
hCD163-R	GTTACAAATCACAGAGACCGCT
hCD206-F	GGGTTGCTATCACTCTCTATGC
hCD206-R	TTTCTTGTCTGTTGCCGTAGTT
hARG1-F	TGGACAGACTAGGAATTGGCA
hARG1-R	CCAGTCCGTCAACATCAAAACT
hIL-8-F	ACTGAGAGTGATTGAGAGTGGAC
hIL-8-R	AACCCTCTGCACCCAGTTTTC

### Preparation of mouse intestinal mucosa

After the euthanasia of mice, a complete colon was obtained. One end of the colon was fixed at the opening of the gavage needle and tied with a sterilized thread for fixation. The colon was then inverted, exposing the intestinal mucosa, which was gently scraped with pliers and placed in tissue protective solution. The operation was on ice throughout the entire process.

### Dimensionality reduction and clustering

Following tissue digestion, samples were sequenced by a 10 × Chromium single-cell platform. We use Seurat to visualize our data. For visualization, Uniform Manifold Approximation and Projection (UMAP) with Seurat functions RunUMAP was used to perform the dimensionality reduction. The PCs used for clustering were the same as those used for calculating the embedding. When calculating UMAP coordinates, the effective ratio of embedded point settings (arrangement) varied from 1 to 2. IrGESA (v 1.1.2) was used to integrate all the single-cell rank-based gene set enrichment analysis.

### Cell–cell communication analysis

Cell–cell crosstalk was inferred by calculating the average expression levels of ligands and receptors in different cell types using the Cell Chat package (version 1.1.3). The mean gene expression of each cell group (negative control group, DSS group and DSS + Fluoxetine group) was calculated based on triMean. Then the mean based on the pathway by summing the communication probabilities of all interactions in each pathway was obtained based on the Kyoto Encyclopedia of Genes and Genomes (KEGG) pathway enrichment analysis. Those with *p*-values < 0.05 were considered to be significant.

### Developmental pseudotime analysis

Monocle was used to conduct developmental pseudotime analysis. First, clustering analysis was conducted with Seurat after cell cycle regression. Then the Seurat object was performed with Monocle, using statistical models to find out differentially expressed (DEG) genes according to the clustering result. Reversed graph embedding (DDRTree method) was used to perform dimensionality reduction. Lastly, a tree-like structure reflecting the developmental trajectories from one cell state to others was performed by manifold learning of Monocle.

### Statistical analysis

Data were analyzed using the statistical software package SPSS 11.0 and R software (version 4.1.2). Groups were compared by one-way analysis of variance (ANOVA) followed by the least significant difference test. *p < 0.05 was considered significant, and **p < 0.01 was considered markedly significant.

## Results

### Study design

**Fig. 1:**
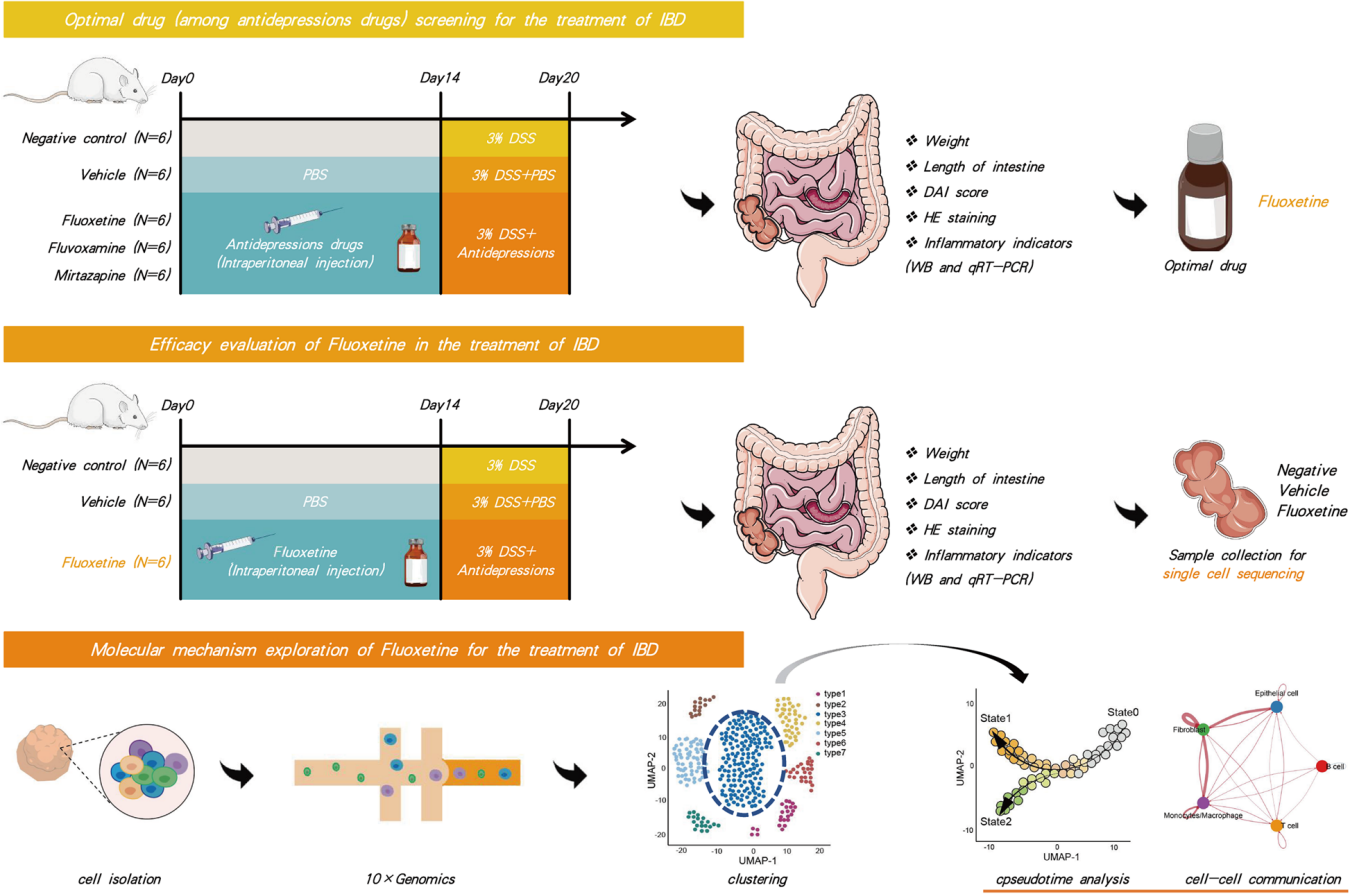
Study design

To simulate the effects of antidepressants on colitis, the normal C57BL/6 mice were intraperitoneally injected with three types of depressions (dissolved in PBS) at 10 mg/kg for 100uL, from day 0 to day 14. Then we treated the mice with 3% (wt/vol) DSS dissolved in drinking water for 7 days, making them similar to human ulcerative colitis (UC) in terms of etiology, clinical symptoms, pathological changes, and treatment response (Fig. 1).

### Antidepressants significantly alleviate colon inflammation

**Fig. 2:**
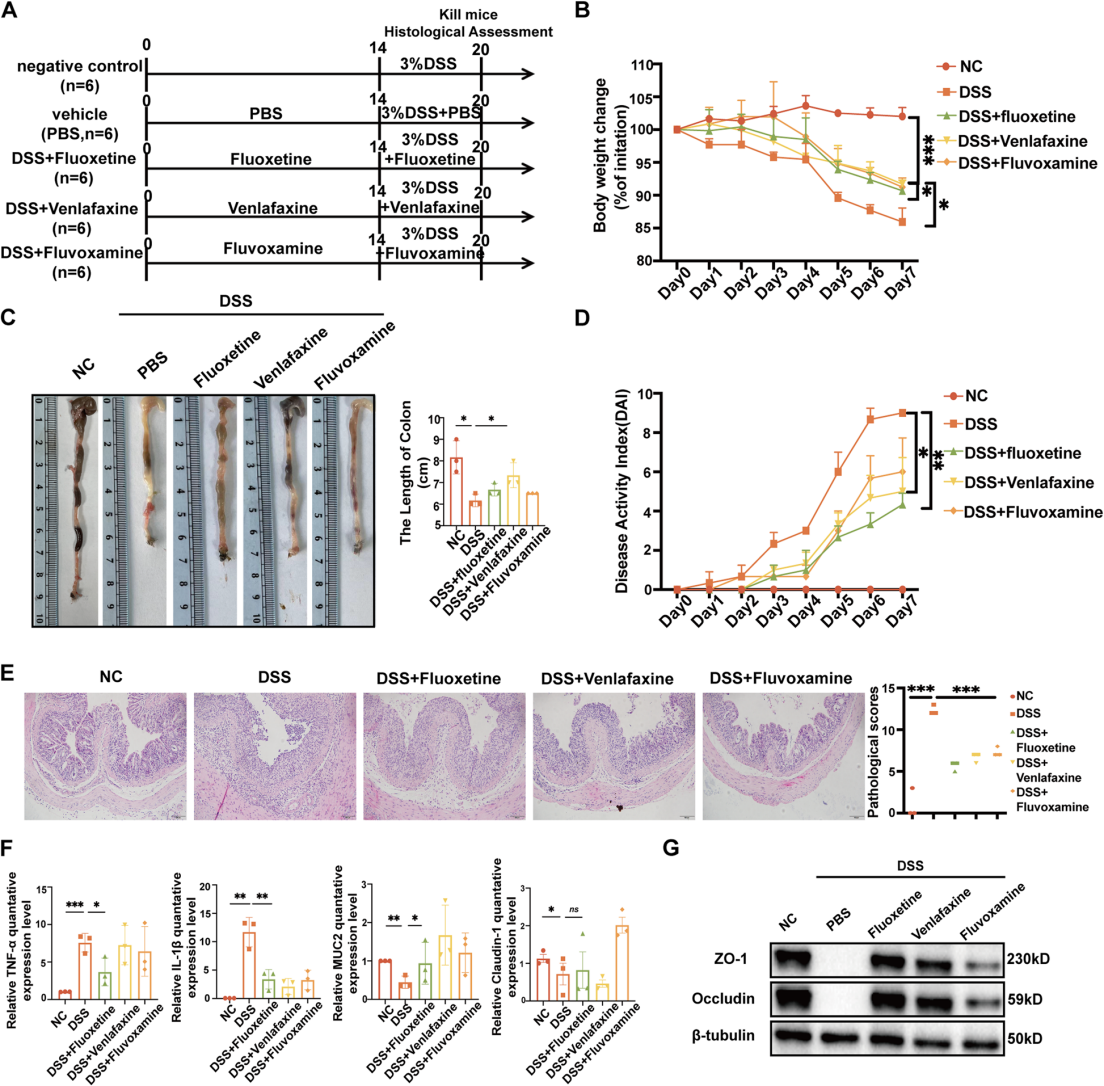
Antidepressants significantly alleviate colon inflammation. **A** Establishment of an ulcerative colitis animal model. The antidepressant groups were intraperitoneally injected with related drug solutions at 10 mg/kg for 100uL, while the DSS group was intraperitoneally injected with an equal volume of PBS. After 14 days, all mice in the DSS group and the antidepressant groups received 3% (wt/vol) DSS dissolved in drinking water for 7 days. **B** Body weight changes. **C** Representative images of the overall appearance and length of the colon in different groups. **D** DAI scores. **E** Representative microscopic images of H&E staining and histology scores. **F** RT-qPCR of the intestinal barrier and inflammation-associated genes. **G** Western-blot results of intestinal barrier-associated genes

All the antidepressants can alleviate body weight loss, increase the colon length and decrease the DAI score. Among them, fluoxetine was the most effective one (Fig. 2A-D). The H&E staining of colon sections in the antidepressant group showed a significant reduction in pathological changes (Fig. 2E). Antidepression treatment downregulated the secretion of anti-inflammatory cytokine TNF-ɑ and IL-1β and upregulated the expression of ZO-1, Claudin-1, and Occludin (Fig. 2 F, G). Thus, we concluded that antidepressants could alleviate the inflammation of the colon, with fluoxetine being the most effective one.

### Fluoxetine significantly alleviates colon inflammation

**Fig. 3:**
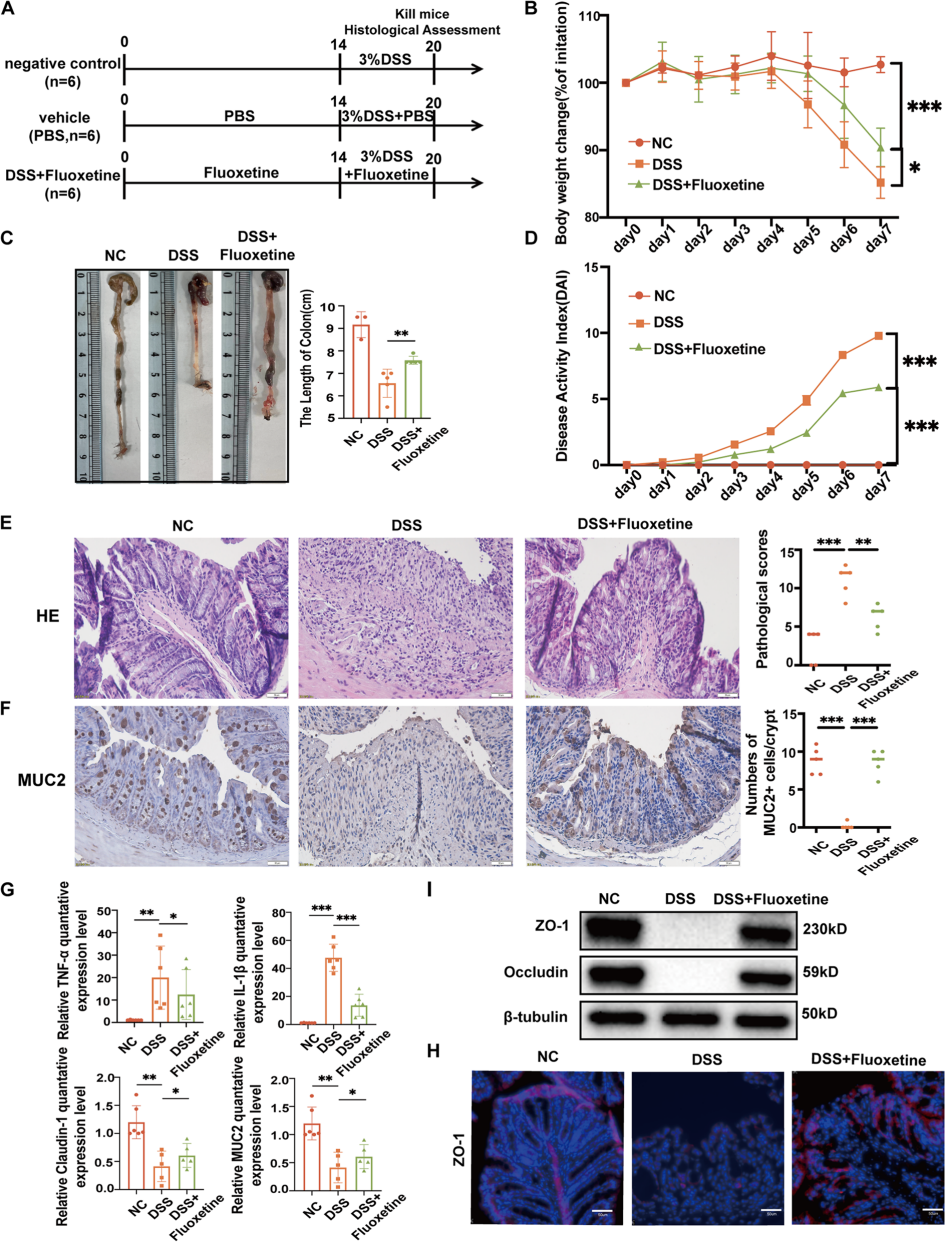
Fluoxetine significantly alleviates colon inflammation. **A** Establishment of an ulcerative colitis animal model. The fluoxetine group was intraperitoneally injected with fluoxetine solution at 10 mg/kg for 100uL, while the DSS group was intraperitoneally injected with an equal volume of PBS. Fluoxetine was dissolved in PBS. After 14 days, all mice in the DSS group and the fluoxetine group received 3% (wt/vol) DSS dissolved in drinking water for 7 days. **B** Body weight changes. **C** Representative images of the overall appearance and length of the colon in different groups. **D** DAI scores. **E** Representative microscopic images of H&E staining and histology scores. **F** Representative images of MUC2 immunohistochemical staining of colonic sections. The scar bar represents 50 μm. **G** RT-qPCR of the intesinal barrier and inflammation-associated genes. **H** Western-blot results of intestinal barrier-associated genes. **I** Representative fluorescent images of ZO-1 staining of colonic sections. Scale bars represent 50 μm

As the most effective drug, fluoxetine was selected for further studies. Fluoxetine successfully alleviated the body weight loss of UC mice, increased the colon length, and decreased the DAI score (Fig. 3A-D). The H&E staining of colon sections in the fluoxetine group showed a significant reduction in pathological changes compared to the DSS group (Fig. 3E). Immunohistochemical staining of MUC2 exhibited a decrease in the DSS group while being restored in the DSS + Fluoxetine group (Fig. 3F). Additionally, fluoxetine treatment downregulated the secretion of anti-inflammatory cytokine TNF-ɑ and IL-1β, while upregulated the expression of ZO-1, Claudin-1, and Occludin (Fig. 3 G, H). IF detection of ZO-1 protein showed that ZO-1 was decreased in the DSS group while rescued in the DSS + Fluoxetine group (Fig. 3I). Together, these results indicated that fluoxetine reduced the secretion of anti-inflammatory cytokine and maintained tight junctions to alleviate the inflammation of the colon.

### Identification of main cell clusters in the mouse intestinal mucosa

**Fig. 4:**
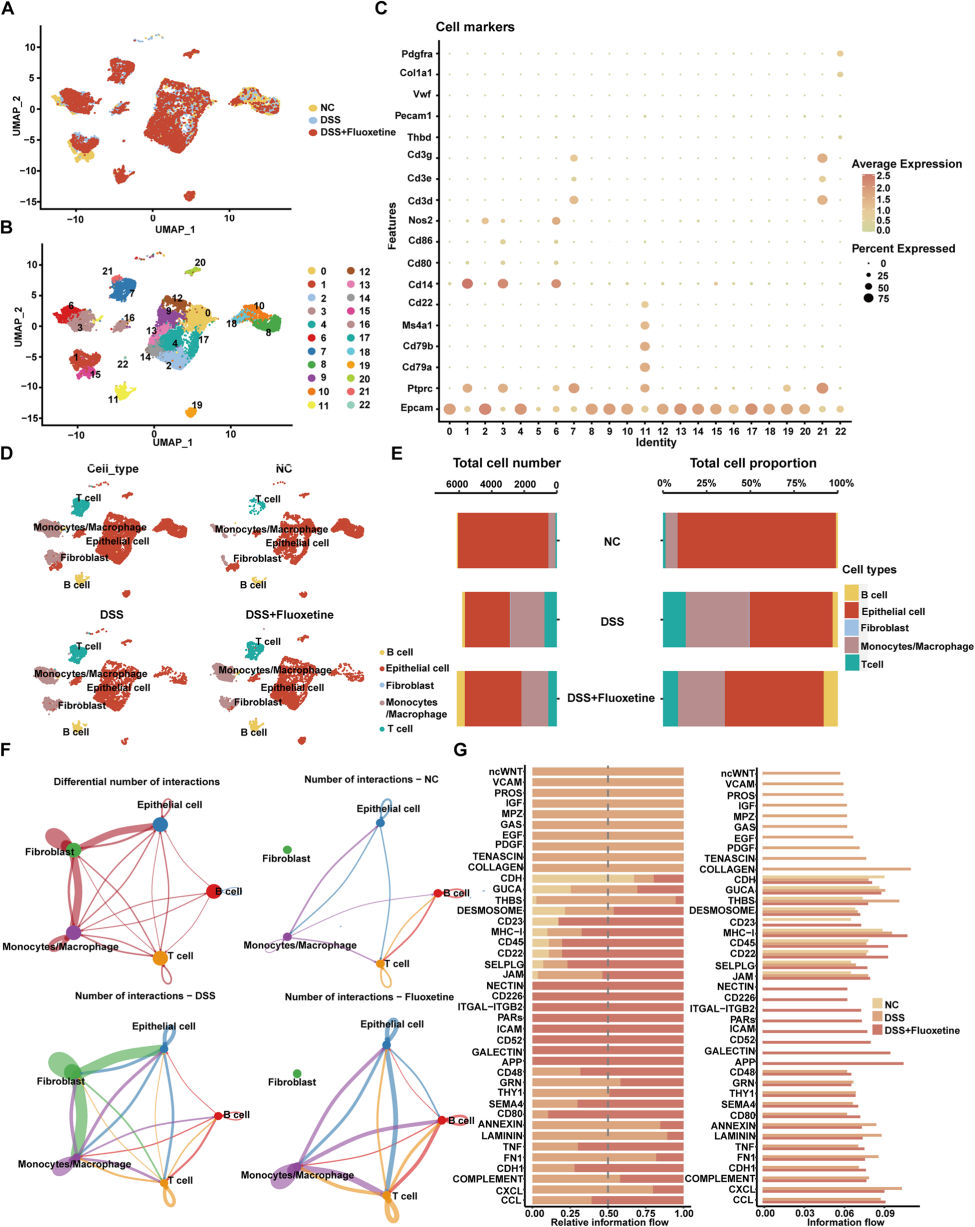
Identification of main cell clusters in the mouse intestinal mucosa. **A** Visualization (a UMAP plot) of all single-cell transcriptomes in mouse intestinal mucosa. **B** A UMAP plot showing 23 clusters and cell population annotations. **C** Bubble heatmap showing marker genes across 23 clusters. **D** A UMAP plot showing 5 total clusters in NC/DSS/DSS + Fluoxetine groups. **E** Total cell number and cell type distributions of the intestinal mucosa in NC/DSS/DSS + Fluoxetine groups. **F** Connected lines represent cell communications between the 5 cell types in the intestinal mucosa in NC/DSS/DSS + Fluoxetine groups. **G** Ligand-receptor pairs analysis based on marker genes in different clusters

To fully assess the effects of fluoxetine on the gut microenvironment, we performed single-cell sequencing analysis on the intestinal mucosa isolated from mice who received fluoxetine and their controls. There were 23 cell clusters identified by the Seurat canonical correlation analysis and visualized with UMAP (Fig. 4A, B). Ptprc was used to distinguish immune and nonimmune cells, and Cd79a, Cd79b, Cd80, Cd86, and Cd3d/e/g were used to further distinguish the specific types of immune cells, including monocyte-macrophages, T cells, and B cells (Fig. 4C). Major cell populations identified including the epithelial cells, monocytes, macrophages, T cells, B cells, and fibroblasts were shown in Fig. 4D. Noticeably, there was an increased monocyte-macrophage and T cell proportion in the DSS group as compared to controls, which was slightly restored in the DSS + Fluoxetine group (Fig. 4E). Cell–cell communication analysis suggested that all 5 clusters had a strong interaction with each other in the DSS group and greatly attenuated in the DSS + Fluoxetine group (Fig. 4F). Ligand-receptor pairs analysis demonstrated that THBS showed higher expression in the DSS group, restoring in the DSS + Fluoxetine group, while Cd45 and Cd22 showed a high level in the DSS + Fluoxetine group compared to the DSS group (Fig. 4G).

### Clustering and pseudotime analysis of epithelial cells from mouse intestinal mucosa

**Fig. 5:**
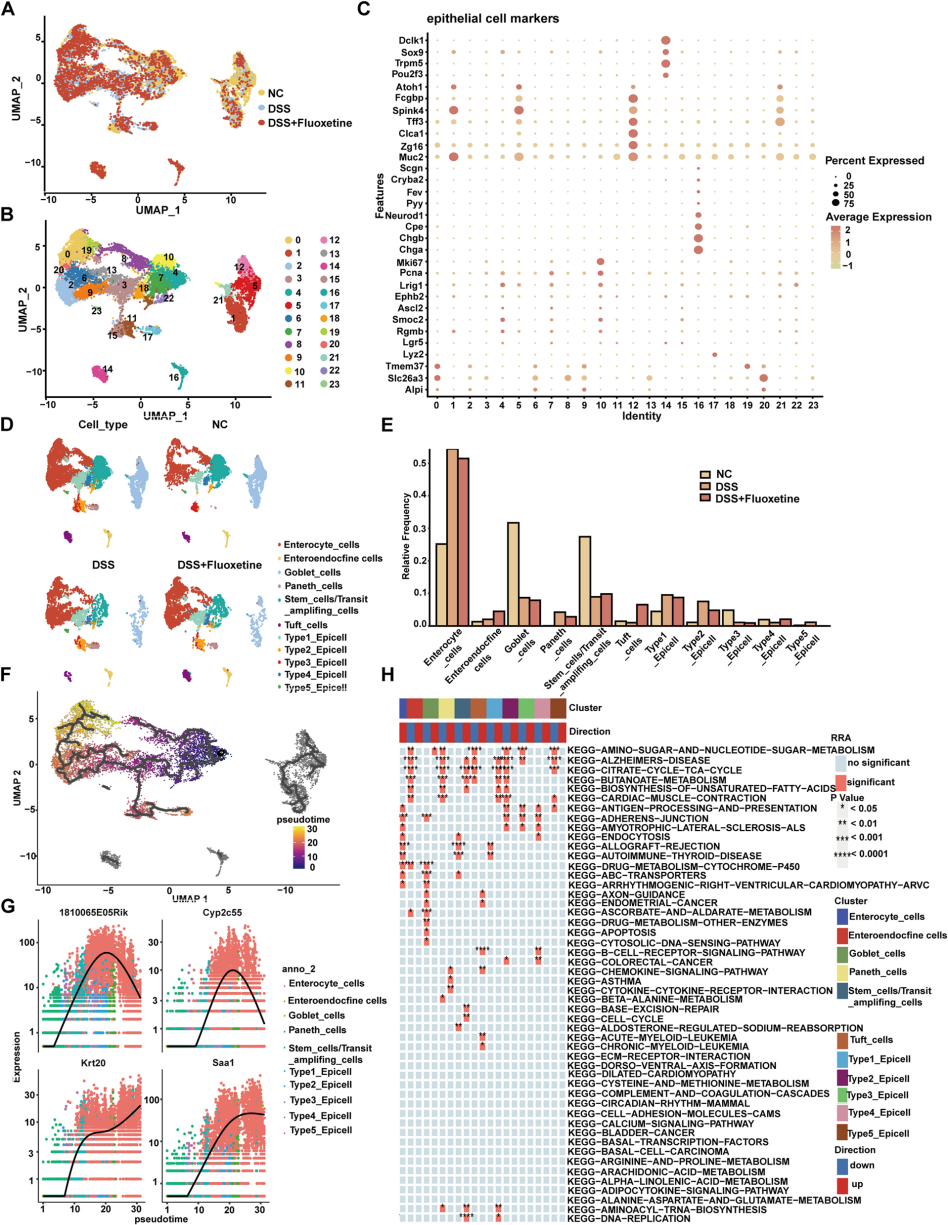
Clustering and pseudoprime analysis of epithelial cells from mouse intestinal mucosa. **A** Visualization (a UMAP plot) of epithelial single-cell transcriptomes in mouse intestinal mucosa. **B** A UMAP plot showing 24 clusters and population annotations of epithelial cells. **C** Dot plots of representative cell type-specific markers. The dot color represents the gene expression level of cells of a cluster expressing the gene; sizes represent the percentage of cells. **D** A UMAP plot showing 11 epithelial cell clusters. **E** Cell proportion distributions of 11 epithelial cell types in the intestinal mucosa of NC/DSS/DSS + Fluoxetine groups. **F** Pseudptime analysis of intestinal epithelial cells showing development trajectories from stem cells to enterocyte cells and paneth cells. **G** Gene expression dynamics for two trajectories in (**E**) along development pseudotime. **H** KEGG enrichment analysis based on marker genes in different clusters

Epithelial cell clusters were further divided into 24 clusters based on Seurat analysis (Fig. 5A, B). Major epithelial cell populations identified included enterocyte cells (cluster 0,2,3,6,8,9,13,19,20), featured by high Alpi, Slc26a3, and Tmem37 levels; the enteroendocrine cells (cluster 16), featured by the expression of Chga, Chgb, Cpe, and Neurod1; the Goblet cells (cluster 1, 5, 12, 21) which showed high expression of Muc2, Zg16, Spink4, and Fcgbp; paneth cells (cluster 17) which exhibited a Lyz2 high level; and stem cells (cluster 4,7,10) with high expression of Mki67, Pcna, and Smoc2 (Fig. 5C, D).

There is a significant difference in the epithelial cell cluster distribution among the 3 groups. Enterocytes, Goblet cells, and stem cells are the three major clusters with similar proportions in the NC group, while enterocytes become the dominating cluster in the DSS and DSS + Fluoxetine groups (Fig. 5E). The pseudotime analysis ordered all epithelial cells along a trajectory from the beginning at stem cells, and bifurcating with 2 branches. Enterocyte cells were located at one end of the trajectory, and paneth cells (along with/differentiated from) type 2 cells were at the other end (Fig. 5F). Expression levels of markers for different clusters were further explored along the pseudotime trajectory (Fig. 5G). Thus, we hypothesized that the branch from stem cells to enterocyte cells represented the differentiation trajectory of the traditional differentiation tract of intestine cells. While more stem cells differentiated to paneth cells through type 2 cells along with the other trajectory after the intake of fluoxetine. Analysis of the trajectory to paneth cells identified upregulated genes toward paneth cells, such as Lyz2, Muc2, and Zg16. Lastly, KEGG enrichment revealed that the cytokine and cytokine-receptor interaction pathway, asthma pathway, and chemokine signaling pathway were enriched among paneth cell clusters, while the citrate cycle tca cycle pathway, cardiac muscle contraction pathway, and adherens junction were inhibited in type 2 epithelial cell clusters (Fig. 5H).

### Clustering and pseudptime analysis of monocyte-macrophages from mouse intestinal mucosa

**Fig. 6:**
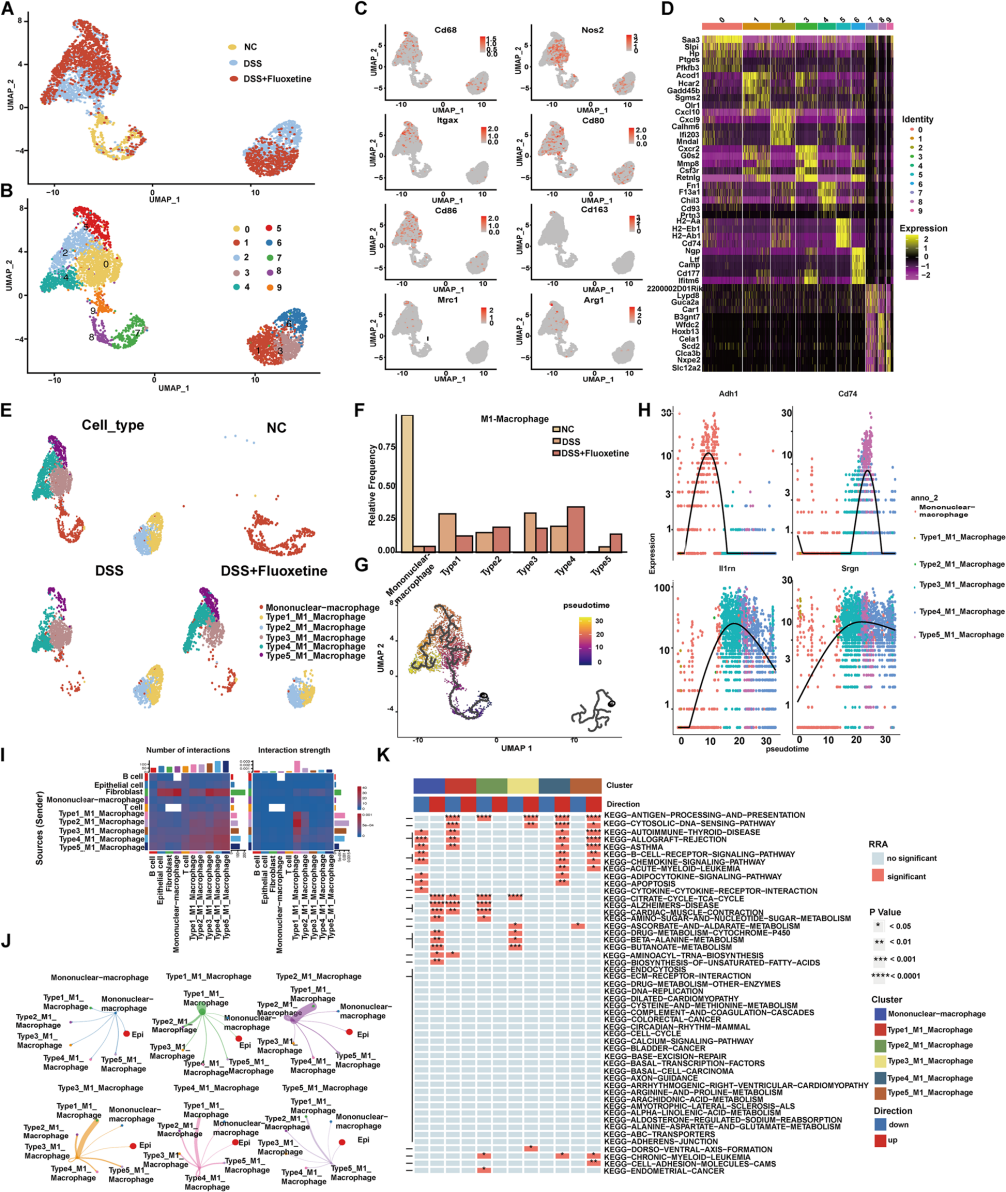
Clustering and pseudoprime analysis of monocyte-macrophages from mouse intestinal mucosa. **A**Visualization(UMAP plots) of monocyte-macrophage single-cell transcriptomes in mouse intestinal mucosa. **B** A UMAP plot showing 10 clusters and population annotations of monocyte-macrophages. **C** A UMAP plot showing the expression of canonical genes among macrophage markers. **D** Heatmap showing the top 20 markers (or all markers if less than 20) (by average log[fold change]) for each of the 10 clusters. **E** A UMAP plot showing 6 monocyte-macrophage clusters. **F** Cell proportion distributions of 9 monocyte-macrophage types in the intestinal mucosa of NC/DSS/DSS + Fluoxetine groups. **G** Pseudptime analysis of monocyte-macrophages showing development trajectories from monocytes to different types of M1 macrophages. **H** Gene expression dynamics for two trajectories in (**G**) along development pseudotime. **I**,**J** Predicted interaction of all the clusters between different types of macrophages. **K** KEGG enrichment analysis based on marker genes in different clusters

Monocyte-macrophage clusters were further divided into 9 clusters based on Seurat analysis (Fig. 6A, B). All the 9 clusters showed high expression of monocyte markers (Cd68, Itgax) and M1 macrophage markers (Nos2, Cd80, Cd86, Arg1) (Fig. 6C, D). The major cell populations identified are shown in Fig. 6E. Among the 5 M1 macrophage clusters, cluster 5 featured high levels of M2 markers (Cd163 and Mrc), showing a trend of M2 polarization. Noticeably, there was an increased cluster 5 in the DSS + Fluoxetine group as compared to the DSS group (Fig. 6E, F). The pseudotime analysis ordered all macrophages along a trajectory from the beginning at monocyte-macrophages, and bifurcating with 2 branches. Type 4 M1 macrophages were located at one end of the trajectory, and type 5 M1 macrophages were at the other end (Fig. 6G). Expression levels of markers for different clusters were further explored along the pseudotime trajectory (Fig. 6H). Thus, we hypothesized that the branch from monocyte-macrophages to M1 macrophages represented the differentiation trajectory of the traditional differentiation tract of macrophages. The complex interactions of various M1 macrophages and other clusters were evaluated by examination of the transcriptomic level of ligands and corresponding receptors (Fig. 6I, J). Finally, KEGG analysis suggested that the autoimmune thyroid disease pathway, cytosolic DNA sensing pathway, and chemokine signaling pathway were enriched among type 5 M1 macrophage cluster (Fig. 6K).

### Fluoxetine promotes M1 macrophages to differentiate into M2 macrophages to significantly alleviate colon inflammation

**Fig. 7:**
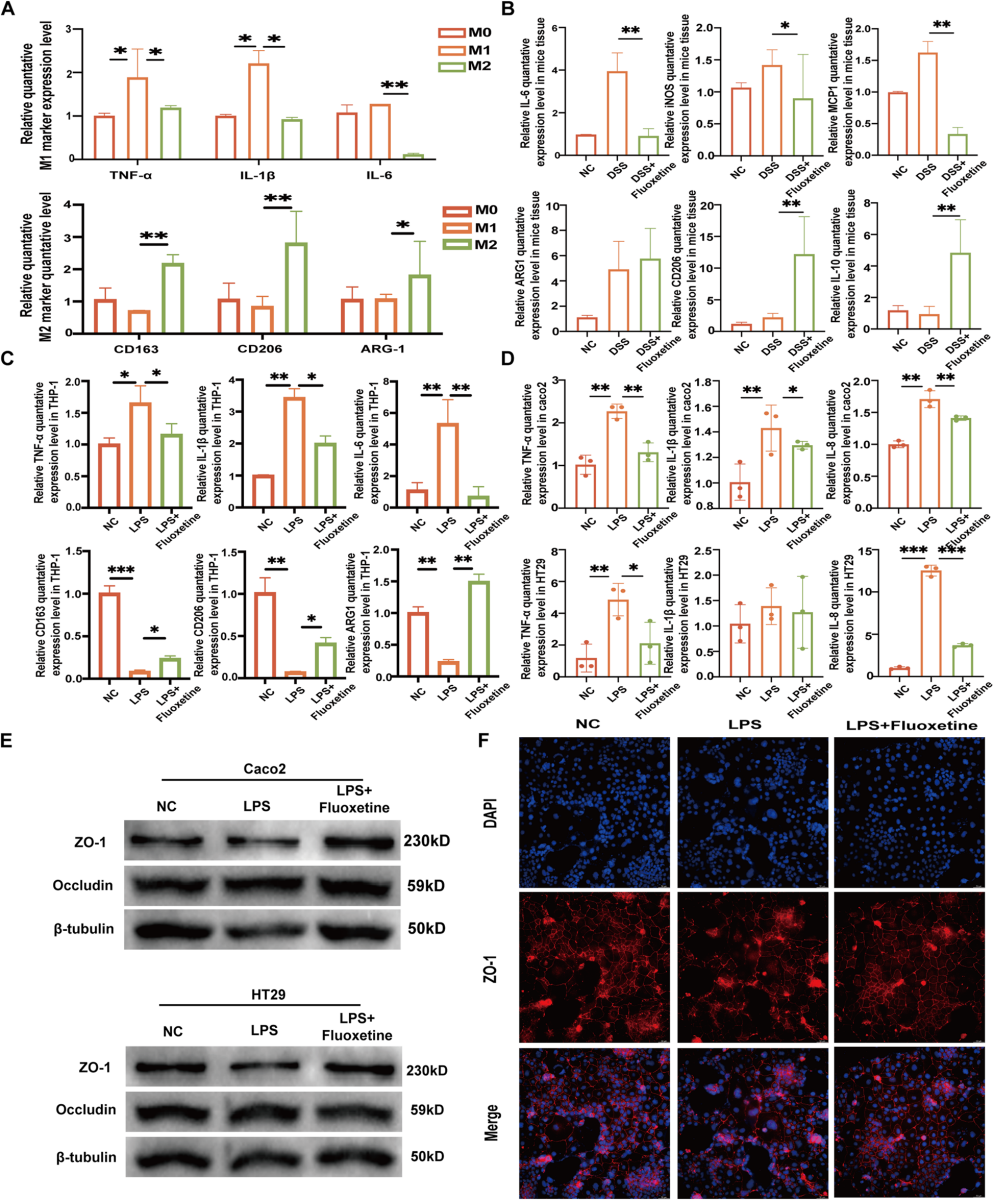
Fluoxetine promotes M1 macrophages to differentiate into M2 macrophages to significantly alleviate colon inflammation. **A** RT-qPCR results of macrophage biomarkers among M0 macrophage, LPS and IFN-γ induced M1 macrophages and IL-4 and IL-13 induced M2 macrophages. **B** RT-qPCR results of macrophage biomarkers in negative/DSSS/DSS + Fluoxetine groups of mice tissue. **C** RT-qPCR results of macrophage biomarkers in negative/LPS/LPS + Fluoxetine groups of THP-1 monocytes. **D** RT-qPCR results of inflammatory-associated genes in negative/LPS/LPS + Fluoxetine groups of Caco2 and HT29. **E** Western-blot results of intestinal barrier-associated genes in negative/LPS/LPS + Fluoxetine groups of Caco2 and HT29. **F** Representative fluorescent pictures of ZO-1 staining of Caco2. Scale bars represent 50 μm

Based on the microenvironment analysis of the mucosa treated by fluoxetine, we proposed that fluoxetine promoted M1 macrophages to differentiate into M2-like macrophages, which exhibited anti-inflammatory effects on enterocytes. Then we tested this hypothesis in vitro. M1 macrophage was induced by the stimulation of LPS and IFN-γ, while M2 macrophage was induced by a combined treatment of IL-4 and IL-13. The qRT-PCR assays demonstrated that DSS/LPS upregulated the level of M1 macrophage markers in both THP-1 (IL-6, TNF-ɑ, and IL-1β) and mice tissue (IL-6, iNOS and MCP-1), while fluoxetine upregulated the level of M2 macrophage markers in both THP-1 (CD163, CD206 and ARG1) and mice tissue (IL-10, CD206 and ARG1) (Fig. 7A-C). To preliminary explore the mechanisms of fluoxetine on macrophages, fluoxetine and tianeptine (a SERT agonist) were used on macrophages. The M2 polarization induced by fluoxetine was rescued by tianeptine (Supplementary Fig. 2). The supernatant obtained from the M1 macrophage treated with LPS upregulated the proinflammatory cytokines, such as IL-8, TNF-ɑ and IL-1β and restored by fluoxetine (Fig. 7D). Similarly, the expression of intestinal barrier proteins (ZO-1 and OCCLUDIN) were significantly destructed in the DSS group and partially restored in the DSS + Fluoxetine group (Fig. 7E, F).

## Discussion

With the deepening understanding of the brain-gut axis [16, 17], it has become a consensus that patients with depression are susceptible to IBD [18]. Our research provided evidence that antidepressants can alleviate the inflammation of IBD. Here, we applied single-cell sequencing to systematically evaluate the effect of antidepressants on IBD in relevant animal models of IBD.

We found that the proportion of intestinal epithelial cells and macrophages in the models of IBD changed significantly. In the further analysis of intestinal epithelial cells, there was a significant change in the number of paneth cells. Previous studies found that paneth cells are the main source of type C lysozyme in intestinal epithelium. Paneth cell lysozyme can balance anti- and pro-inflammatory responses of the intestine, with an impact on IBD [19]. There are two broad hypotheses to explain this phenomenon. Firstly, the drug directly inhibits the production of paneth cells, exerting a protective effect on IBD. Secondly, the inflammation of mice was alleviated under the treatment of fluoxetine. The proportion of paneth cells in the low inflammatory state decreased as a result of the negative feedback effect. Our research creates new possibilities for identifying an innovative therapeutic approach to treat IBD.

A novel finding in the present study is the effect of fluoxetine to induce macrophages to M2-like phenotype. However, one limitation of our study is that the macrophages with higher M2 markers were derived from M1 macrophages or monocytes is still unclear. More research needs to be conducted in the future to achieve a better understanding of the differentiation process of macrophages, such as the mechanisms dependent on 5-hydroxytryptamine (5-HT) or not. Our data revealed that the macrophages stimulated by fluoxetine exerted an anti-proinflammatory effect on the model of IBD, which is consistent with the previous studies about the effect of M2-like macrophages on IBD[20.21]. Our data reveals that immune cells like macrophages may play an important role in the treatment of fluoxetine on IBD.

It’s known that the vagus nerve plays an important part in the tonic inhibition of acute inflammation in IBD, which is an important part of the brain-gut axis. Depression interfered with the tonic vagal inhibition of proinflammatory cells and increased susceptibility to intestinal inflammation and tricyclic antidepressants reduced intestinal inflammation by restoring vagal function [20]. Some other studies also found that depression reactivated dormant chronic colitis depended on the α7 subunit of the nicotinic acetylcholine receptor (α7nAchR), which normalized upon treatment with antidepressants [21]. Notably, our research found that antidepressants may play a certain direct role in intestinal epithelial cells and immune cells in the mucosal microenvironment with no dependence on the vagus nerve, which is a supplement to the vagal nerve-dependent mechanism.

As we all know, the main target of fluoxetine is the 5-HT transporter (SERT), which blocks reuptake and prolongs neurotransmitter signaling of 5-HT [22]. In the gut, response to serotonin is mediated via a wide repertoire of serotonin receptors which exist in various cells, including immune cells and cancer cells [23]. It has been proved that the stimulation of 5-HT can promote M2 macrophage polarization, but inhibit M1 macrophage polarization [24].

Other research has reported that the activation of 5-hydroxytryptamine receptor 4 located in intestinal epithelial cells maintains healthy colon motility in mice and guinea pigs, and alleviates inflammation in the colon of mice with colitis [25]. This is consistent with the results of our findings in the changes in these cells. Our data has found that fluoxetine has a specific direct effect on macrophages and intestinal epithelial cells. However, it is still unclear whether the direct impact of fluoxetine on protecting the intestinal mucosa depends on the SERT or not. Additional transporters or receptors may be essential to the function of fluoxetine on intestinal mucosa.

We have identified the function of fluoxetine and other antidepressants in the treatment of IBD, but there are also some limitations. First, due to the stable blood concentration and duration of administration, we could only evaluate the preventive effects on IBD. Previous studies [26–28] suggested that fluoxetine could also have a preliminarily therapeutic effect on IBD. Future studies need to be done to investigate the preliminarily therapeutic effect of fluoxetine on IBD. Second, large animal models close to human size need to be studied in the future. Third, effects on the microbiome have not been evaluated as part of this study. We revealed the possible mechanisms underlying the effect of antidepressants on IBD, which can guide drug development and comprehensive care for patients with IBD and psychiatric comorbidities. This provides a new perspective on the drug development of IBD.

## Conclusions

Fluoxetine exhibited anti-inflammatory effects on intestinal mucosa via remodeling of the intestinal cells and macrophages, which reveals that fluoxetine is a promising therapeutic drug for the treatment of IBD and psychiatric comorbidities.

### Supplementary Information

**Additional file 1.**

**Additional file 2:** **Figure S2.** RT-qPCR results of macrophage biomarkers in negative/LPS/LPS+Fluoxetine/LPS+Fluoxetine+Tianeptine groups of THP-1 monocytes.

## Data Availability

Raw data of this article will be available from the corresponding author at [wujing36youyi@ccmu.edu.cn] upon reasonable request.

## Abbreviations

IBD: Inflammatory bowel diseases
UC: Ulcerative colitis
DSS: Dextran Sulfate Sodium
LPS: Lipopolysaccharides
DAI score: Disease Activity Index
UMAP: Uniform Manifold Approximation and Projection
H&E: Hematoxylin and eosin
TNF-ɑ: Tumor Necrosis Factor-alpha
IL-1β: Interleukin-1 beta
ZO-1: Zona Occludens 1
MUC2: Mucin 2
IFN-γ: Interferon-gamma
IL-4: Interleukin-4
IL-6: Interleukin-6
IL-8: Interleukin-8
IL-10: Interleukin-10
IL-13: Interleukin-13
iNOS: Inducible Nitric Oxide Synthase
Arg1: Arginase-1
MCP-1: Macrophage chemoattractant protein-1
CD163: Cluster Of Differentiation163
CD206: Cluster Of Differentiation206
