# Supplementary figures and images for: Antidepressant fluoxetine alleviates colitis by reshaping intestinal microenvironment
Source: Cell Commun Signal. 2024 Mar 12;22:176. doi: 10.1186/s12964-024-01538-5 (PMC10935910; doi:10.1186/s12964-024-01538-5)

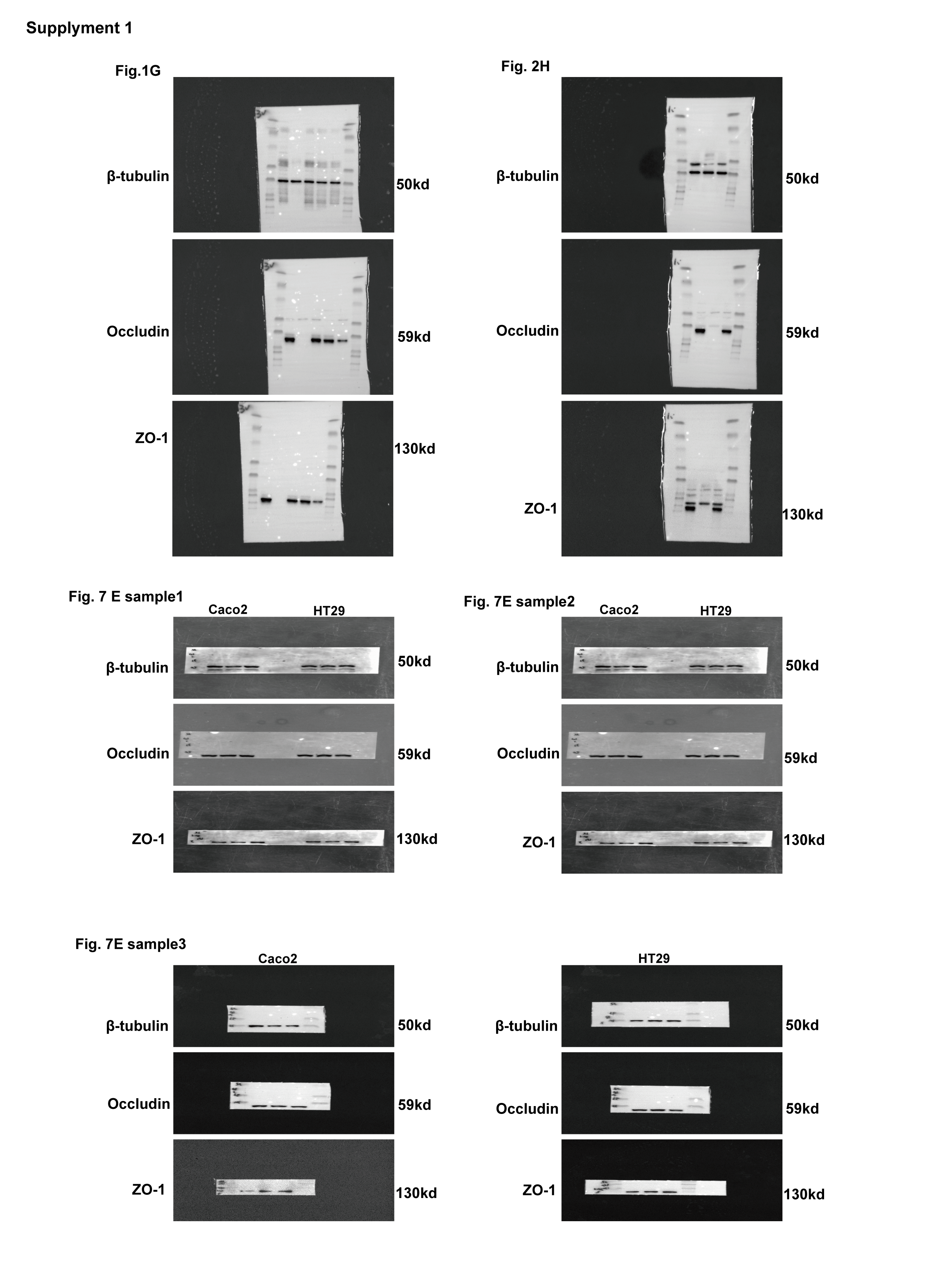

Supplement: Supplementary file 1 — Additional file 1. [file 12964_2024_1538_MOESM1_ESM.tif]

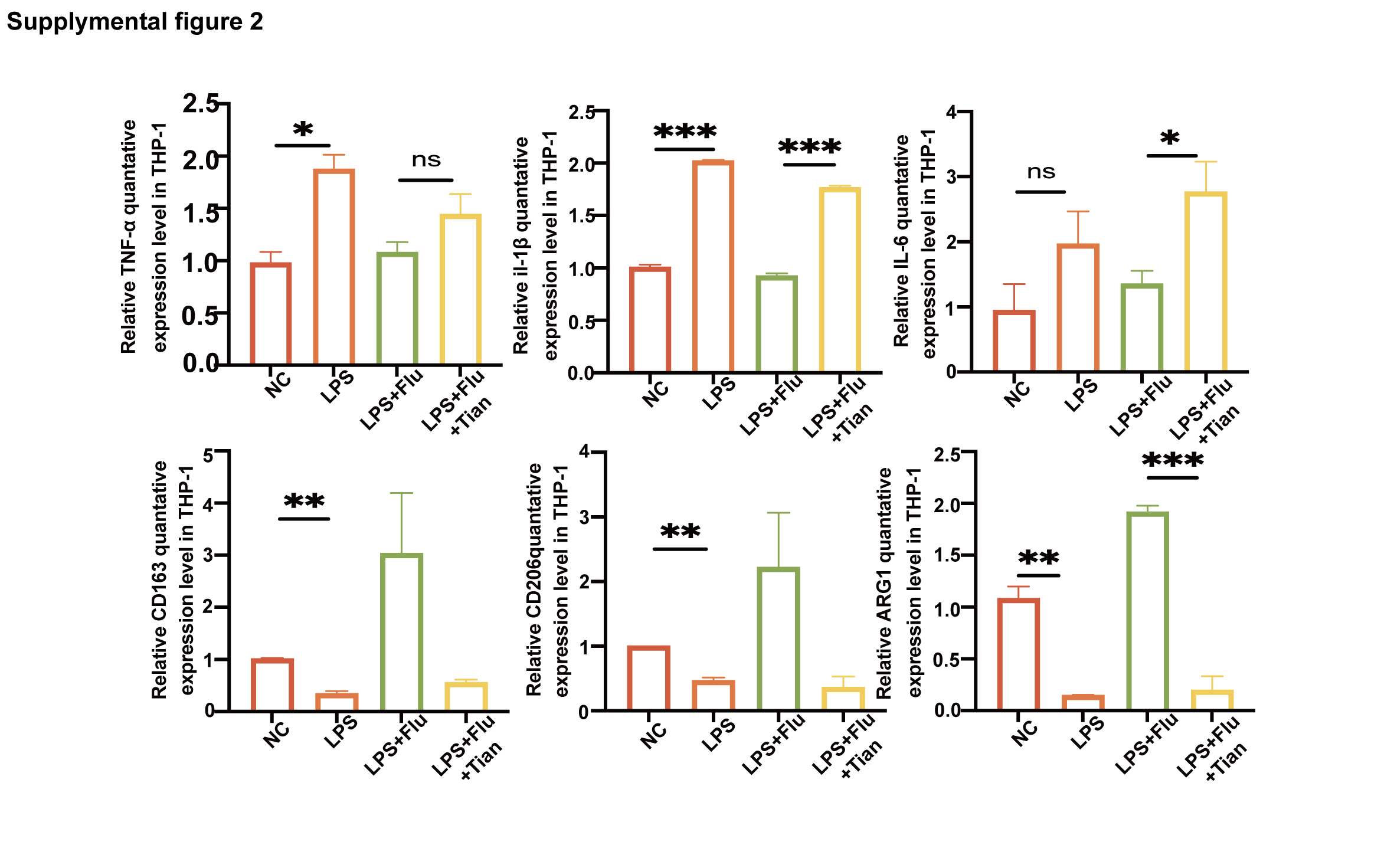

Supplement: Supplementary file 2 — Additional file 2: Figure S2. RT-qPCR results of macrophage biomarkers in negative/LPS/LPS+Fluoxetine/LPS+Fluoxetine+Tianeptine groups of THP-1 monocytes. [file 12964_2024_1538_MOESM2_ESM.tif]
